# Seasonal influences on sleep and executive function in the migratory White-crowned Sparrow (*Zonotrichia leucophrys gambelii*)

**DOI:** 10.1186/1471-2202-11-87

**Published:** 2010-07-29

**Authors:** Stephanie G Jones, Elliott M Paletz, William H Obermeyer, Ciaran T Hannan, Ruth M Benca

**Affiliations:** 1 Department of Psychiatry, University of Wisconsin, Madison 6001 Research Park Blvd, Madison, WI 53719, USA

## Abstract

**Background:**

We have previously shown that the White-crowned Sparrow (WCS) decreases sleep by 60% during a period of migratory restlessness relative to a non-migratory period when housed in a 12 h light: 12 h dark cycle. Despite this sleep reduction, accuracy of operant performance was not impaired, and in fact rates of responding were elevated during the migratory period, effects opposite to those routinely observed following enforced sleep deprivation. To determine whether the previously observed increases in operant responding were due to improved performance or to the effects of migration on activity level, here we assessed operant performance using a task in which optimal performance depends on the bird's ability to withhold a response for a fixed interval of time (differential-reinforcement-of-low-rate-behavior, or DRL); elevated response rates ultimately impair performance by decreasing access to food reward. To determine the influence of seasonal changes in day length on sleep and behavioral patterns, we recorded sleep and assessed operant performance across 4 distinct seasons (winter, spring, summer and fall) under a changing photoperiod.

**Results:**

Sleep amount changed in response to photoperiod in winter and summer, with longest sleep duration in the winter. Sleep duration in the spring and fall migratory periods were similar to what we previously reported, and were comparable to sleep duration observed in summer. The most striking difference in sleep during the migratory periods compared to non-migratory periods was the change from discrete day-night temporal organization to an almost complete temporal fragmentation of sleep. The birds' ability to perform on the DRL task was significantly impaired during both migratory periods, but optimal performance was sustained during the two non-migratory periods.

**Conclusions:**

Birds showed dramatic changes in sleep duration across seasons, related to day length and migratory status. Migration was associated with changes in sleep amount and diurnal distribution pattern, whereas duration of sleep in the non-migratory periods was largely influenced by the light-dark cycle. Elevated response rates on the DRL task were observed during migration but not during the short sleep duration of summer, suggesting that the migratory periods may be associated with decreased inhibition/increased impulsivity. Although their daily sleep amounts and patterns may vary by season, birds are susceptible to sleep loss throughout the year, as evidenced by decreased responding rates following enforced sleep deprivation.

## Background

Migratory songbirds appear to have an unprecedented ability to withstand the effects of sleep loss during the migratory period. In the wild, despite marked reductions in apparent opportunity to sleep, they continue to engage in adaptive waking behaviors including prolonged flight, complex navigation, and predator evasion and foraging in novel environments. In the captive White-crowned Sparrow (*Zonotrichia leucophrys gambelii)*, we have previously shown that during periods of migratory restlessness, sparrows reduced sleep duration by an average of 60% relative to the non-migratory state [1], a finding supported in other avian species [1-4]. Despite this apparent sleep reduction, migratory sparrows showed no deficits in learning or performance in a standard operant task when compared to non-migrating sparrows. Given that cognitive and performance deficits are some of the most consistent consequences of sleep loss in mammals [5], the preservation of neurobehavioral performance seems surprising. However, are all aspects of executive function intact during the migratory period and what are the separate or distinct contributions played by migratory state and/or sleep duration in these executive function processes?

In our previous study, we reported that during the migratory period birds maintained the ability to learn a new sequence of key pecks each day and were consistently able to execute a previously learned sequence of key pecks at a level comparable to that observed during the non-migratory periods [1]. However, although accuracy of operant performance was not impaired by migratory status, we did observe that rates of responding were significantly higher during the migratory period relative to the non-migratory period. Increased response rates in this context ultimately proved adaptive since they were concomitant with increased food reinforcement and allowed the migrating birds to meet their higher caloric needs. However, in this operant schedule, it was not possible to determine whether the elevated response rate during the migratory period was a consequence of a greater motivation to work for food reward or related to a general increase in activity. Here we sought to determine whether birds in the migratory state will increase operant responding whether that responding is adaptive or maladaptive.

Our previous observations of sleep and operant behavior in the White-crowned Sparrow were made during one fall migratory period and one late summer non-migratory period. Birds were housed under photoperiodic conditions of 12 h:12 h LD, so that the observed changes were attributable to endogenous migratory factors rather than to the combined effects of migratory status and a seasonally changing photoperiod. However, not only do the seasonal changes in day length affect the organization and timing of avian migration [6,7], these changes also have substantial effects on sleep duration outside of the migratory period in the few avian species that have been studied under natural conditions [8-10]. In captive European Starlings (*Sturnus-vulgaris*) housed under naturalistic photoperiodic conditions, sleep duration in early spring is ~10% shorter than that observed in mid-winter [9]. During mid-winter, when the dark period is 16 hours, the captive Rook (*Corvus frugilegus*) was shown to sleep ~37% of the 24 hour period, whereas during the short 7 hour dark period of summer, sleep occupied only ~20% of the 24 hour period [8]. Thus, in at least some avian species, decreases in sleep duration closely parallel seasonal increases in day length. Under naturalistic conditions, it is likely that daily sleep amounts in the White-crowned Sparrow change not only in response to migratory status, but also to seasonal changes in daylight exposure.

In this study we assessed the effects of the light-dark cycle and migratory status on sleep patterns in the White-crowned sparrow and examined the contributions of migratory status and sleep duration on operant performance. Specifically, we have characterized the electrophysiological correlates of sleep for one 24 hour period during four distinct seasons-winter, spring, summer and fall-using an adjusting, species-specific photoperiod designed to simulate the lighting conditions typically experienced in the wild by the WCS. Operant performance was assessed in these same four seasons using a differential-reinforcement-of-low-rate-behavior (DRL) procedure, a task in which optimal performance depends on the bird's ability to withhold a response for a fixed interval of time; elevated response rates ultimately impair performance by decreasing access to food reward. The DRL procedure is routinely used to assess behavioral inhibition, or its converse, impulsivity [11].

## Methods

### Subjects

White-crowned Sparrows (*Zonotrichia leucophrys gambelii*) were captured in California (Sutter and Colusa counties) between March 2004 and March 2005. For the assessment of sleep a total of 13 birds were recorded, 12 of which were ultimately analyzed for sleep/wake scoring (SLPs). One was analyzed in three seasons (Summer, Fall and Winter), two were analyzed for two seasons (Spring and Summer) and the remaining 9 were analyzed for one season each. 15 DRL birds (DRLs) were assessed for 4 seasons after birds had learned the task, defined as stable performance (4 consecutive sessions with response rate for each session varying by no more than 10% of the mean response rate across all 4 of those sessions), before entering a migratory season. Therefore, each bird was studied for 2 non-migratory seasons and 2 migratory seasons. Migratory birds do not display activity patterns consistent with frank migratory activity for the whole of the spring or fall. However, for the purposes of this study, unless stated otherwise, birds selected from the spring or fall were always in the active migratory state and displaying activity patterns which typify this state, including increased nocturnal activity. Similarly, birds in the summer and winter are always in the non-migratory state. All birds were captured using mist nets under authorization granted by the California Department of Fish and Game and the United States Fish and Wildlife Service.

Sparrows were transported to the University of Wisconsin - Madison where they were individually housed in galvanized wire cages (L: 35 cm × W: 25 cm × H: 32 cm) in environmentally controlled rooms (L: 4.0 m × W: 2.7 m × H: 2.7 m; 22.0 - 24.5°C, 40% relative humidity). Each bird was in visual and auditory contact with other birds in the room. Their daily diet consisted of a seed mixture (Finch Mix, Mounds Pet Food Warehouse, Middleton, WI), grit, romaine lettuce, and one mealworm. The SLPs had access to food and water *ad libitum*. DRLs had their food restricted for 3 hours per day (see Behavioral Procedures), except on weekends, and access to water was not available during 30-minute test sessions. All procedures were approved by the Institutional Animal Care and Use Committee at the University of Wisconsin - Madison and followed NIH guidelines. The study was conducted in an AAALAC-accredited facility.

### Photoperiods

The photoperiods in housing and testing rooms approximated seasonal and geographic changes in day length appropriate for these birds based on their typical location. Dawn and dusk times were changed each Friday evening to reflect the day length at the expected location of *Z.l. gambelii *on that day of the year. Winter and summer photoperiods were based on US Naval Observatory (http://aa.usno.navy.mil./data) sunrise/sunset tables for Sacramento, CA and Fairbanks, AK, respectively. Spring and fall photoperiods were based on a linear approximation of a direct route between Sacramento and Fairbanks and observed arrival and departure dates [12]. Illuminance during the dark phase was < 0.5 lux. Illuminance during the light phase was 540 - 640 lux measured at the level of the cage floor. Photoperiods for days selected for sleep scoring were as follows: spring 14.75:9.25 LD, summer 21.5:2.5 LD, fall 13.75:10.25 to 16.0:8.0 LD and winter 9.5:14.5 LD. Bird behavior was more variable during the fall, so that recordings for birds in this group were obtained on different days, with correspondingly different lengths of simulated daylight. Operant data were analyzed from birds under the same photoperiodic conditions as above, except in fall when photoperiods ranged from 11.5:12.5LD to 16.0:8LD.

### Activity monitoring

Selection of days for sleep scoring from each season was partly based on data from activity monitoring. To measure daily activity, an infrared (IR) photocell sensor (Invisible-Beam Entry Alert, Radio Shack, Fort Worth, TX) was centered behind each bird's home cage, 18 cm from the floor and 17.5 cm from either side of the cage. The sensor was positioned to project the photobeam parallel to and above two perches so that hops and flights from one side to the other disrupted the beam. Beam breaks were tallied every 30 seconds (i.e., interval recording, with 2880 intervals per day) using VitalView (version 4.0) software and transmitter equipment (Mini Mitter Co., Inc., Bend, OR). Although the infrared activity monitoring system may be prone to some inaccuracy because it is insensitive to activity unless the bird crosses the center-line of the cage and overly sensitive to activity when the bird remains near the center of the cage and on the perches, it nevertheless offers a rapid method for assessing gross seasonal changes in behavior. SLPs behavior was also continuously recorded using two infrared-sensitive cameras per bird connected to a digital video storage system (IView PC, Salient Systems Corp., http://www.salientsys.com). Infrared illuminators provided lighting for the cameras during the dark phase. Cameras were positioned on opposite sides of each cage, to afford maximum potential for visual monitoring of behavior. Cameras (commonly available for recording and transmitting over the Internet) were used inside the test chambers to view DRLs during sessions.

### Surgery

Only SLPs received surgery. Surgical procedures were performed under isoflurane anesthesia (1.0%-3.5% isoflurane with 500 ml/min O2). The bird's head was stabilized in a stereotaxic device (Kopf Instruments, Tujunga, California, United States), cranial feathers were removed and an incision was made along the midline of the head to expose the cranium. Six small holes were drilled through the cranium to the dura: Two holes were drilled on each hemisphere of the anterior forebrain 2 mm lateral to the midline over the hyperpallium (Wulst). Two additional holes were drilled 2 mm posterior to the anterior holes so that signal was recorded from both the anterior and posterior hyperpallium. Two holes were positioned over the midline of the cerebellum to accommodate the common reference electrode and the ground. Teflon insulated stainless steel electrodes (#791400, A-M Systems Sequim, Washington, United States) were inserted through the holes to the level of the dura and held in place with surgical adhesive (Tissuemend II, Veterinary Products Laboratories, Phoenix, Arizona, United States).

Each electrode was connected to a lightweight, flexible, and electrically shielded recording cable designed for use with small birds [13] (Dragonfly, Inc., Ridgeley, West Virginia, United States). This cable was attached to the skull using dental acrylic (Justi Products, Oxnard, California, United States), and the incision was closed around the acrylic with surgical adhesive (Tissuemend II, Veterinary Products Laboratories, Phoenix, Arizona, United States). Following surgery, each bird was placed in the recording cage and provided with at least 14 days of postoperative recovery and adaptation to the recording cable before experimental observation began.

### Electrophysiological recording

The recording cable was attached to a low torque 6-channel mercury commutator and the weight of the recording cable was counterbalanced with a spring; these recording conditions allowed birds to move unimpeded throughout the cage. The EEG signals were referenced to the cerebellar electrode, amplified and band pass filtered (0.3 - 30 Hz and 10 - 90 Hz, respectively) using Grass-telefactor amplifiers (Model 12 Neurodata and 7P511, http://www.grass-telefactor.com) and digitized at 100 Hz (National Instruments PCI 6071E card, http://www.ni.com and Somnologica 2, Flaga hf. Medical Devices, http://www.medcare.com). EEG signals were viewed using Somnologica 3 software (Flaga hf. Medical Devices, http://www.medcare.com).

### Sleep-wakefulness scoring

**Figure 1:**
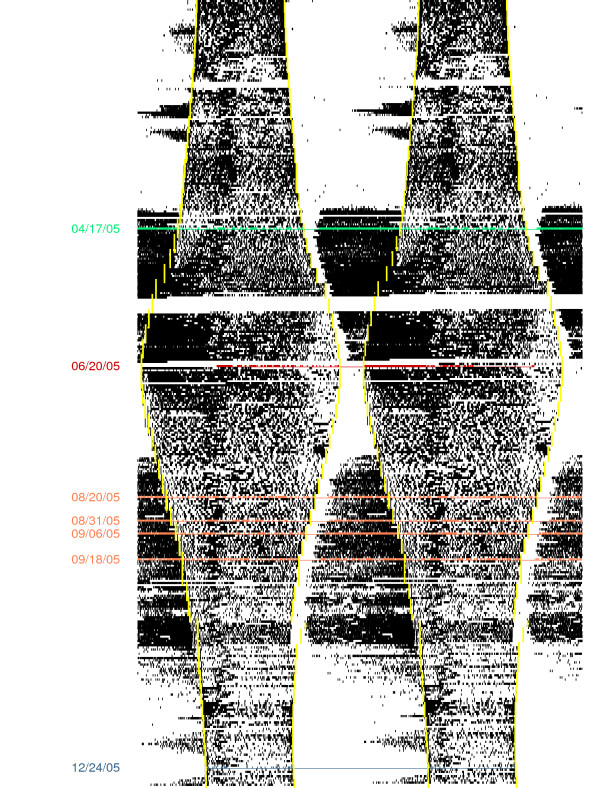
**This double plotted actogram shows activity for a representative bird over the course of approximately 30 months, including 2005 when the sleep data for this paper were collected**. Vertical yellow lines indicate seasonal changes in daylight. Seasons from which birds were selected for sleep analyses are highlighted (winter (blue) n = 4, spring (green) n = 5, summer (red) n = 4, fall (brown) n = 3). Photoperiods for the winter, spring, summer, and fall seasons were, respectively, 9.5:14.5 LD, 14.75:9.25 LD, 21.5:2.5 LD, and 13.75:10.25 to 16.0:8.0 LD.

Actograms (e.g., see Figure 1) generated from the IR activity data demonstrated that daytime activity was moderate to high in all seasons. Migratory-specific behavior was best characterized by nighttime IR beam-breaking activity. Blocks consisting of several consecutive days of nocturnal activity were selected for each bird. Video footage was reviewed to ascertain that video quality on the day selected for scoring was adequate. To assess extremes of sleep-wakefulness behavior during non-migratory seasons, days for scoring were chosen during the longest (summer) and shortest (winter) photoperiods. Days were also selected during spring and fall migratory periods when nocturnal activity appeared maximal. We eliminated days for which video recordings were not consistent with IR-measured activity, e.g., a bird slept perched near the middle of the cage and generated many IR beam-breaks with small involuntary movements. The EEG and video recordings were reviewed concurrently to ascertain EEG quality, ensuring that the EEG was not obscured by motion artifact (outside of active wakefulness) and that the quality of the tracing was sufficient to distinguish among vigilance states (wakefulness, drowsiness, SWS, REM sleep). For each of the seasons of winter, spring and summer, we were able to find at least a single date that met these criteria for all the birds being recorded at those times. Fall migratory behavior was more variable within and between birds, both in timing and in day-to-day consistency, than spring migratory behavior, as previously reported [14]; this necessitated the use of data from different days for different birds.

During each season, 4 or 5 birds produced 24-hour records (activity, video, and EEG) that fit our selection criteria, resulting in a total of 16 scored records across four seasons. Unfortunately, data for individual birds could not be collected across all seasons, as EEG signals typically degraded within 3 - 9 months, and, consequently, 10 of the 13 birds were recorded during one season only; 2 birds were recorded for two seasons and 1 was recorded for three seasons. One fall bird originally selected for sleep scoring was ultimately not used in our final data analysis; although IR data indicated that this bird was in the migratory state on the date selected to score his sleep, he was not, on closer inspection of the video, actively engaged in nocturnal migratory activity, and no other migratory days for this bird had EEG data of usable quality. As a result, only 3 fall birds were used in the final data analysis.

Vigilance state was manually scored in 4 s epochs using simultaneous EEG and video recordings. Each epoch was categorized as either wakefulness, drowsiness, SWS or REM sleep based on visual inspection of the EEG from both hemispheres, as well as by analysis of recorded behavior using the standard criteria: Wakefulness was characterized by a high-frequency low-amplitude EEG in both hemispheres. Behavior during wakefulness included hopping and flying around the cage, feeding, drinking, feather preening, and actively scanning the room. During drowsiness, EEG activity was intermediate between that of wakefulness and SWS (i.e., increased amplitude in the low-frequency range relative to wakefulness). Behavioral evidence of drowsiness included birds holding their heads close to their bodies and the position of the eyelids fluctuating between open, partially closed, and completely closed states. During SWS, EEG activity was dominated by slow waves of the highest amplitude. Behaviorally birds were motionless, with closed eyes; the head was either pulled in toward the body and facing forward or resting on the bird's back. REM sleep epochs in birds tend to be brief [15]. In this study, a 4-sec epoch was scored as REM sleep if the EEG amplitude was reduced by at least one-half the amplitude seen in the previous SWS episode and this amplitude reduction lasted for longer than 2 seconds and was accompanied by behavioral signs of REM sleep including muscle hypotonia (feather or head drooping) or, rarely, eye movements. Bouts of sleep were defined as the length of time from a SWS onset to the next bout of wakefulness (W), and waking bout length was likewise defined as the length of time from the onset of W to an epoch of SWS. We also calculated the ratio of sleep bout length to waking bout length as a measure of relative sleep stability for each hour. This measures whether sleep is maintained for longer periods of time than wakefulness. For each 24-hour period, the entire period was scored in 4 s epochs, resulting in 21,600 epochs per day.

### Operant Apparatus

Programs for operant behavior testing and data collection were written using MED-PC IV (Med-Associates, St. Albans, VT). Session events were recorded with 10-millisecond resolution. Behavioral testing was conducted in operant chambers (Med-Associates, Model ENV-007). Each chamber contained a translucent pigeon key capable of emitting white light and a food hopper that, when activated, emitted light and provided 5 seconds of access to nyger seed. The key was situated in the center of the panel with the food hopper located to the right of the key. Each chamber was surrounded by a sound-attenuating cubical with a built-in ventilating fan that circulated air into the experimental environment and provided masking noise. A fluorescent light with an 8-watt bulb, which remained on throughout the experiment, was located between the chamber and cubicle to provide additional lighting. Internet cameras were also mounted inside the chamber cubicles.

### Operant testing

Food was removed from home cages for 3 hours preceding test sessions. This was done Monday through Friday, although sessions were only conducted Tuesday through Friday every week. Following training, operant testing was conducted for 1 year. Sessions were held once per day and each lasted 30 minutes. Each session began with the darkened key turning white. A differential-reinforcement-of-low-rate (DRL) schedule was in effect throughout the entire session [11]. On the DRL schedule used here, a response was reinforced when it occurred more than 20 seconds after the previous response. Qualifying key pecks immediately opened the reinforcement hopper for 5 seconds; response rates per minute were determined by subtracting out this 5 second reinforcement interval. Each key peck that occurred after 20 seconds or more since the last key peck (DRL 20-sec) was reinforced with food. Qualifying key pecks turned the key dark and simultaneously opened the reinforcement hopper for 5 seconds, followed by the key light turning white again; responses made prior to the required 20 second pause had no effect on the key light but did reset the DRL clock to zero to begin incrementing again. Responses during times when the key was dark had no programmed consequences. The DRL schedule is alternatively referred as reinforcement of long interresponse times (IRTs)[11].

Video was monitored to verify that birds were actually consuming the reinforcer when obtained. Training for the procedure began in the fall for 5 birds, in the spring for 2 birds, and in the summer for 8 birds. For all birds, key pecking behavior came under reliable control of the DRL schedule, as defined above, within 6 months.

### Sleep Deprivation

During each season, a 48-hour sleep deprivation was conducted with all DRLs using the following procedure: Members of the research staff entered the housing room once every 3 - 5 minutes or sooner if behavioral signs of sleep, such as eye-closings and inactivity, were prominent (viewed remotely with video cameras). Walking quietly past the cages and occasionally tapping on them provided sufficient stimulation to keep the birds awake; handling was not necessary to induce wakefulness. The sleep deprivation began at lights out on Monday, and continued until lights out on Wednesday evening. DRL sessions were run from Tuesday-Friday each week. Therefore, the first two sessions for the week (Tuesday and Wednesday) occurred under conditions of sleep deprivation; the sessions conducted on Thursday and Friday were considered recovery days. The week prior to the sleep deprivation (Pre Sleep Deprivation) was used as a baseline, and the week subsequent to the sleep deprivation and recovery was used as a return to baseline (Post Recovery). For spring, summer and winter, sleep deprivation was conducted 1 - 2 weeks after the weeks used in the assessment of typical DRL performance for the season. However, during fall, the sleep deprivation probe occurred 1 - 4 weeks after the weeks selected to represent seasonal performance for 10 birds; given; the heterogeneity of the fall migratory behavior, birds used for the sleep deprivation and subsequent DRL testing (post sleep deprivation recovery) in fall were not in the migratory state. The photoperiods (and weeks) during the four sleep deprivation periods were 10.25:13.75 LD for winter (second week of February), 17.25:6.75 LD for spring (second week of May), 20.25:3.75 LD for summer (third week of July), and 13.25:10.75 for fall (fourth week of September). Pre Sleep Deprivation and Post Recovery data were averaged across all 4 sessions of those weeks; Sleep Deprivation and Recovery were averaged across the first 2 sessions and last 2 sessions, respectively, of that week.

### Statistics

Based on selection criteria (see Activity monitoring and Sleep-wakefulness scoring), seasons for SLPs were defined as follows: winter 9.5:14.5 LD (first week of January), spring 14.75:9.25 LD (third week of April), summer 21.5:2.5 LD (first week of July), and fall ranging from 13.75:10.25 to 16.0:8.0 LD (fifth week of August to second week of October). These weeks included those with the longest or shortest days (winter and summer), and those with the most nocturnal activity in the home cage (spring and fall). The main effects and interactions of season, lights and migration status on vigilance state were assessed by repeated measures (split plot) ANOVA. All pairwise comparisons were assessed with a familywise α = 0.05 via Tukey HSD following a significant independent ANOVA.

For DRLs, data from these same photoperiods were used to characterize performance with exception of fall; in this case, the week during which nocturnal home-cage activity (see Activity monitoring) best represented migratory restlessness was selected for each bird. Fall weeks for DRLs fell within the range for SLPs specified above.

For analyses of operant behavior, the primary dependent measures were (1) response rate, defined as the ratio of the number of responses to the number of minutes the key light was on, (2) number of food reinforcers obtained, and (3) the behavioral inhibition ratio (sometimes referred to as efficiency), defined as the ratio of the number of reinforcers to the number of responses. For the behavioral inhibition measure, a ratio of 0 represents complete disinhibition; the closer the ratio is to 1.0, the more pronounced the inhibition. On occasion, birds did not respond during the session; for these sessions, the behavioral inhibition ratio, but not response rate or reinforcers obtained, was dropped because the resultant ratio of 1.0 was not representative of performance. Data were assessed across four consecutive seasons for each bird, starting with the first season of reliable responding (see Operant testing). DRL data were averaged across sessions for each season, producing a single value for each photoperiod before it changed.

For analyses of DRL performance by migratory status, data from the migratory seasons were averaged and compared to non-migratory season averages. T-tests were used in these analyses with the Type I error rate set at 0.05; multiple comparisons were not made following these omnibus tests. For analyses of seasonal performance, a repeated measures ANOVA was performed across all four seasons." Behavioral changes within sessions were also assessed by dividing each session into ten 3-minute bins and recording the number of responses and reinforcers during each bin. Analyses of trends were conducted following repeated measures ANOVAs across the ten bins to determine linearity. For each season of sleep deprivation, a repeated measures ANOVA was performed across conditions of Pre Sleep Deprivation (Baseline), Sleep Deprivation, Recovery, and Post Recovery, followed by post-hoc t-test comparisons with the error rate corrected via the FDR procedure.

## Results

We characterized sleep and operant behavior across a full year using species-location-specific exposure to a changing photoperiod. In Figure 1, a double plotted actogram shows activity for a representative bird over the course of approximately 30 months, including 2005 when the sleep data for this paper were collected. Colored horizontal lines indicate the calendar weeks from which experimental birds were selected. The changing duration of the photoperiod across seasons is indicated by the vertical yellow lines. Daytime activity is shown within the yellow lines, and nighttime activity outside these lines. During winter and summer, activity is completely confined to the light period (area within the yellow lines). In contrast, during spring and fall migratory restlessness, activity extends into the nocturnal period.

Four behavioral states were reliably distinguished based on visual inspection of the EEG and behavioral analysis: wakefulness, drowsiness, SWS and REM sleep. Although the placement of the EEG electrodes was constructed to detect interhemispheric EEG asymmetries, no episodes of unihemispheric sleep were observed. Given the relatively small differences in EEG activity between wakefulness and SWS in the bird, visual scoring of the EEG alone often results in a failure to detect all but the most extreme examples of interhemispheric asymmetries. Furthermore, despite use of several cameras, we could not observe eye closure state 100% of the time, depending on the location of the bird in the cage. Thus, it cannot be ruled out that our failure to detect such asymmetries could be due to technical limitations. However, if any unihemispheric sleep occurred, its duration was likely minimal. Another possible explanation for the absence of unihemispheric sleep in the present study is that this behavior was not expressed because it had no immediate adaptive value under our housing conditions.

**Figure 2:**
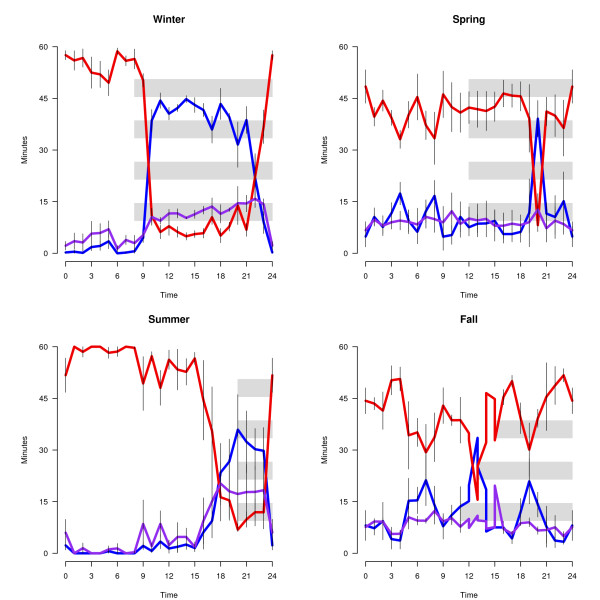
**Time course of each vigilance state, wake (red), sleep (purple) and drowsiness (purple), in each of the four seasons**. Values plotted as mean number of minutes (± SEM,) of each state in 1-hour intervals. Time 0 represents lights on. Note that the fall birds were not scored on the same day and so each bar indicates the dark period for one of the three birds.

**Figure 3:**
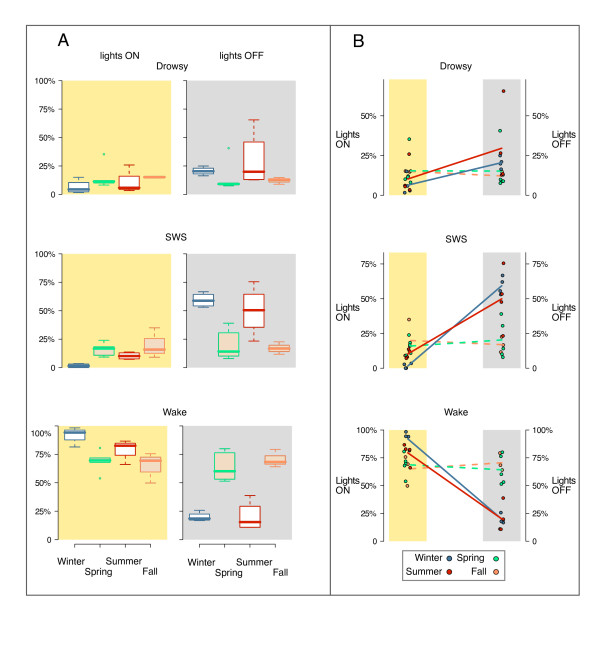
**States of vigilance as a function of light and dark**. Figure 3A. Box-and-whisker plots comparing percentage of time spent in each vigilance state: Drowsy, Sleep (SWS+REM) and Wake across the seasons, plotted separately for light (yellow background) and dark (gray background). Horizontal lines within boxes denote medians (50^th ^percentile); horizontal edges of the boxes denote the 25^th ^and 75^th ^percentiles; whiskers extend to the 5^th ^and 95^th ^percentiles. Open symbols show the outliers. Figure 3B. States of vigilance as a function of light and dark in the four seasons. The percentage of time in each state is shown (colored circle) for each bird in the light (yellow background) and in the dark (gray background). The seasonal average of all birds is plotted at the endpoints of the lines. Lines highlight the change in vigilance state expression from light to dark during winter (blue) and summer (red), and the relative lack of change in spring (green) and fall (brown). Note that the length of the dark period varies with the season so equal percentages do not reflect equal times.

Table 1 shows average percentage (mean ± SEM) of 24-hour recording time spent in each behavioral state across season (top), as well as the number of hours birds spent in each vigilance state and the corresponding length of daylight (bottom). In the lower panel REM has been added to SWS and labeled as "sleep", since REM sleep amounts were minimal throughout. Consistent with our previous findings, overall sleep time was reduced in the two migratory periods combined (fall and spring) when compared to the two non-migratory periods combined (summer and winter) (p < 0.05, Tukey HSD). However, sleep duration in winter and summer are markedly influenced by the duration of the nocturnal period (total daily sleep amount in winter is significantly greater than in the summer), (p < 0.05, Tukey HSD); the longer daylight period in the summer results in sleep duration comparable to that observed in both migratory periods. Figure 2 describes the time course of sleep, wakefulness and drowsiness across the day for each season during the dark and light periods. During the two non-migratory periods, sleep and wakefulness are organized by the light/dark cycle; sleep is largely confined to the dark and wakefulness is preferentially expressed in the light. However, sleep in non-migratory summer is consistently initiated shortly before lights-off, whereas in non-migratory winter, sleep is initiated shortly after lights-off. In the two migratory periods, however, the organizing effect of the light/dark cycle on the distribution of sleep and wakefulness is no longer as evident. Specifically, as shown in Figure 3A, during the two migratory seasons, sleep is significantly reduced in the dark period (p < 0.05, Tukey HSD) and significantly increased in the light (p < 0.05, Tukey HSD) relative to sleep in the two non-migratory seasons. Similarly, wakefulness is significantly increased in the dark (p < 0.05, Tukey HSD), and significantly decreased in the light (p < 0.05, Tukey HSD) during the two migratory seasons. Figure 3B highlights the change in sleep-wakefulness expression from light to dark in the non-migratory periods, and the relative lack of change in the migratory periods. In the two non-migratory seasons, as expected, sleep is significantly more likely to occur in the dark than it is to occur in the light (p < 0.05, Tukey HSD) and wakefulness is significantly more likely to occur in the light than the dark (P < 0.05, Tukey HSD). In contrast, during the migratory seasons, the percent of time spent in sleep or the percent of time spent awake does not change as a function of lighting condition. Waking behavior also differed qualitatively in light vs. dark periods during migration, as previously described [1]. Birds at night engaged in behaviors typical of migratory restlessness (e.g., wing whirring and beak up flight simulation); these behaviors did not occur during the daytime during migration or at any time during the non-migratory days analyzed.

**Table 1:** Overall sleep time in the White-crowned sparrow was reduced in migratory periods and in periods of longer daylight

	Percent			
Season	Wake	Drowsy	SWS	REM
Winter	48.3%	14.9%	36.6%	0.168%
	± 2.2%	± 0.6%	± 2.2%	0.020%
Spring	66.8%	15.3%	17.8%	0.108%
	± 4.5%	± 5.6%	± 3.3%	0.029%
Summer	73.1%	12.3%	14.6%	0.068%
	± 4.4%	± 6.0%	± 2.3%	0.035%
Fall	67.1%	14.0%	18.8%	0.147%
	± 5.6%	± 0.5%	± 5.1%	0.047%

	**Hours**			
**Season**	**Wake**	**Drowsy**	**Sleep**	**Daylight**

Winter	11.6	3.58	8.8	9
	± 0.54	± 0.15	± 0.53	
Spring	16.02	3.67	4.28	13
	± 1.2	± 1.07	± 0.8	
Summer	17.53	2.96	3.51	21
	± 1.05	± 1.44	± 0.57	
Fall	16.13	3.36	4.56	14.29
	± 1.21	± 0.13	± 1.38	± 1.02

(Top) Average percentage (mean ± SEM) of 24-hour recording time spent in each behavioral state across season. (Bottom) Number of hours per 24-hour recording time that birds spent in each vigilance state and the corresponding length of daylight. REM has been added to SWS and labeled as "sleep", since REM sleep amounts were minimal throughout. (winter n = 4, spring n = 5, summer n = 4, fall n = 3).

**Figure 4:**
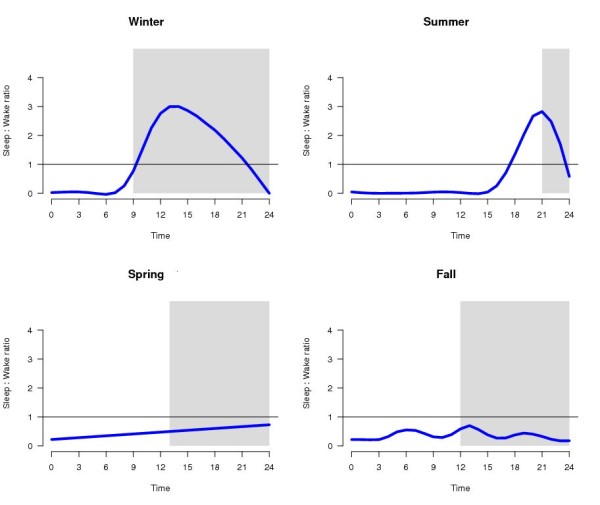
**Sleep consolidation index for all seasons**. Hourly average ratio of sleep bout length to wake bout length was calculated for birds during each season. The blue line represents a smoothed curve through these data. The time during which the smoothed value of the ratio of sleep bout length to wake bout length exceeded 1.00 was used to define the "sleep consolidation index" for each season. In winter, hour 10 is first hour in which the ratio exceeds 1 and this ratio falls below 1 at hour 21, resulting in a sleep period of 11 hours. In summer, the sleep period begins at hour 18 and ends at hour 23. It is noteworthy that this sleep period begins prior to the onset of darkness. A similar phenomenon can be observed in the sleep times plotted in Figure 2. There was no sleep consolidation during the migratory seasons, spring and fall.

Figure 4 shows the relative stability of sleep (i.e., the ratio of sleep bout length to waking bout length) and demonstrates the extent to which the propensity of sleep maintenance was, or was not, preferentially confined to a discrete period within the 24-hours in each season. During the spring and fall migratory conditions, there is no period in the day during which the mean sleep bout duration exceeded the waking bout duration. Furthermore, the relationship between the likelihood of extending a sleep bout and the likelihood of extending a wake bout was biphasic in the non-migratory seasons and generally less organized during the migratory seasons. Note that the smoothed curves in Figure 4 do obscure a brief rise in the relative stability of sleep at about hour 20-21 in the spring and fall. In contrast, in non-migratory winter and summer, there is a clear, consolidated period of sleep, defined as the time during which the smoothed average sleep stability was greater than 1.0. The duration of these periods is markedly different in each non-migratory season. Specifically, in summer, the typical sleep period begins approximately 80 minutes before lights off and lasts for 5 hours. The initiation of sleep before lights off in summer is likely a consequence of the extremely short nocturnal period in this season (2.5 hours). In winter, the sleep period begins approximately 60 minutes after lights off and continues for 11 hours. Daily sleep to wake transitions by season (sleep-drowsy-wake transition treated as a single transition from sleep to wake) did not significantly differ between any seasons or pair of seasons. Numbers of awakenings averaged 271 (± 41) in winter, 205 (± 25) in spring, 193 (± 24) in summer, and 388 (± 55) in fall. Although fall showed the largest number of awakenings, there was not a significant difference between fall and any of the other seasons (nor between any other pair of seasons) on this dimension. The order (fall, winter, spring, summer) by number of awakenings does not speak strongly to hypotheses about the effect of migratory state or season.

### Operant Behavior

**Figure 5:**
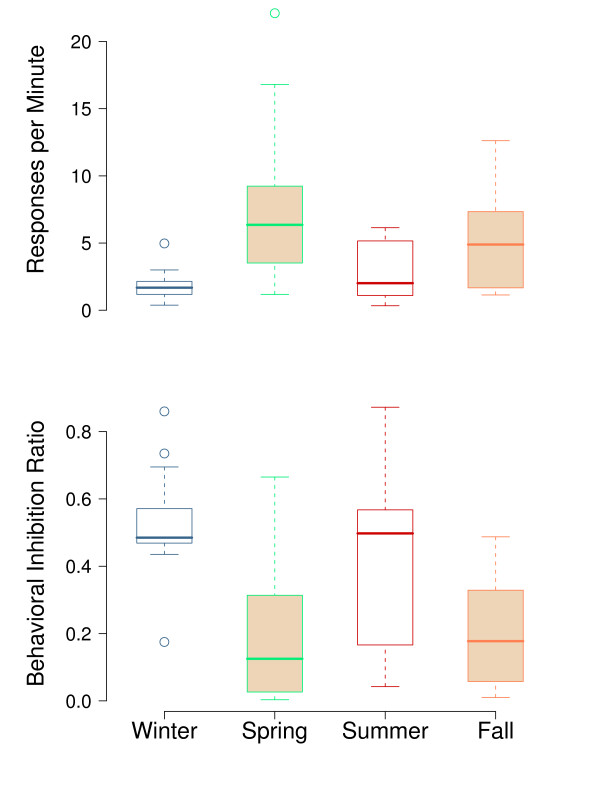
**Box-and-whisker plots illustrating DRL performance during each season**. Top shows response rate (responses/minute) and bottom shows the behavioral inhibition ratio (reinforcers/responses). Horizontal lines within boxes denote medians (50^th ^percentile); horizontal edges of the boxes denote the 25^th ^and 75^th ^percentiles; whiskers extend to the 5^th ^and 95^th ^percentiles. Open symbols show the outliers. *N *= 15. Response rates are significantly higher during the migratory periods relative to the non-migratory periods and the behavioral inhibition ratio was significantly lower during migration. Operant data were analyzed for the same weeks, except in fall when operant animals were exposed to photoperiods ranging from 16.0:8LD - 11.5:12.5LD.

In DRL procedures, the extent to which an animal makes a premature response, and consequently reduces the number of rewards obtained, putatively reflects impulsivity [11]. Figure 5 shows box plots for response rate (the ratio of the number of responses to the number of minutes the key light was on; top), and the behavioral inhibition ratio (the ratio of the number of reinforcers to the number of responses; bottom), during each of the migratory and non-migratory seasons. A bird performing the task perfectly should achieve a behavioral inhibition ratio of 1; the lower the ratio, the greater the failure to inhibit behavior. Data for individual subjects were averaged across sessions for each week (or range of weeks) shown in Figure 1. In general, birds produced lower rates of responding during non-migratory winter and summer and higher rates during migratory spring and fall. In non-migratory periods, average response rates fall within the expected range of 3 responses per minute. During fall and spring migratory seasons, however, average response rates are significantly elevated (t(14) = 3.94, P = 0.001) relative to the two non-migratory seasons. As a result, the behavioral inhibition ratio was significantly lower in the two migratory seasons (t(14) = -6.61, P < 0.001) relative to the two non-migratory seasons. Despite having previously learned to execute the 20 second delay, during the migratory fall and spring this ability was impaired.

**Figure 6:**
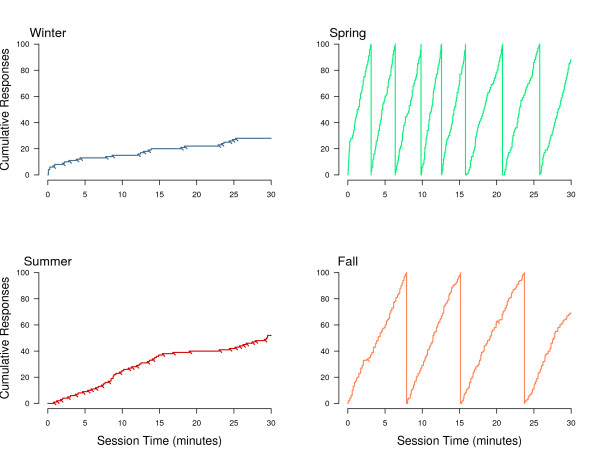
**Cumulative records for an individual bird from a single session during each season**. Diagonal lines extending directly beneath the record (hash marks) indicate food reinforcement. Note there are no reinforcements earned during the spring season. Vertical lines extending from 100 to 0 responses (spring and fall only) are resets of the "pen" due to high rates of responding; this was done to keep the scale constant across each of the four records.

**Figure 7:**
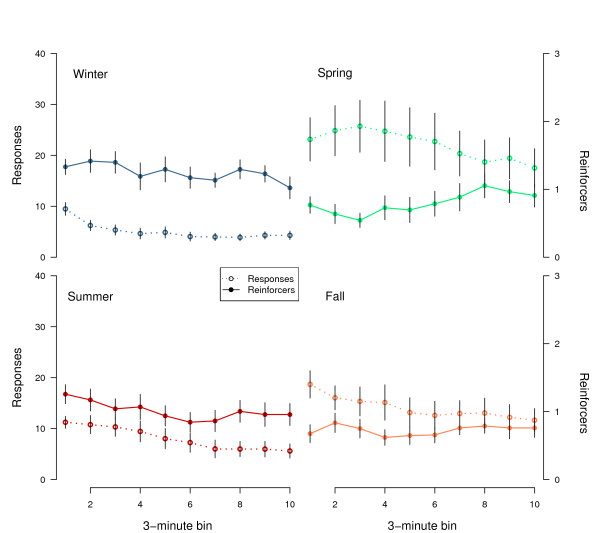
**Performance within sessions on the Differential Reinforcement of Low Rate (DRL) task**. Mean ± SEM (n = 15) of number of responses (left axis, dotted lines) and number of reinforcers (right axis, solid line) are plotted for each 3 minute period in the 30 m session.

**Figure 8:**
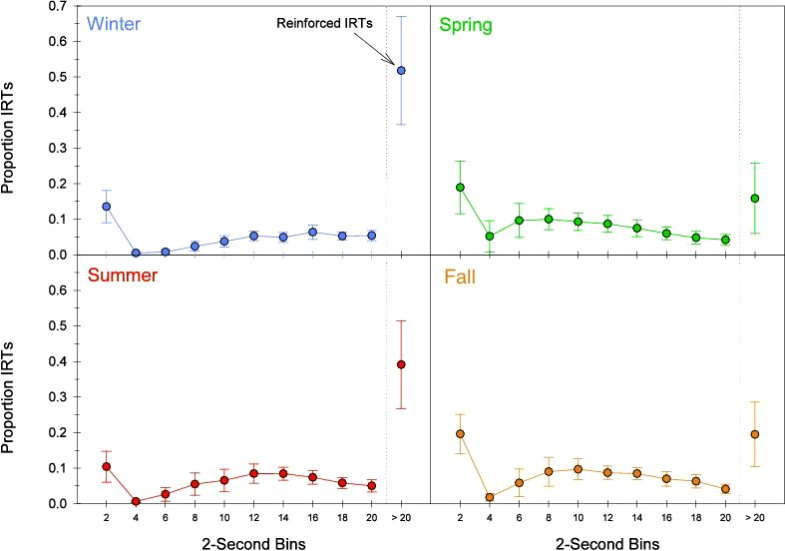
**Relative frequency distribution of interresponse times (IRTs) using a bin size of 2 seconds averaged across birds in each season**. Dashed line indicates the time at which IRTs were reinforced. Proportion of reinforced IRTs, those which occur after the 20 second delay, is shown to the right of the dashed line. IRT distributions are typically bimodal, with the most IRTs either short or long enough to be reinforced. *N *= 15. Error bars represent the 95% CI.

Figures 6, 7, 8 further characterize changes in operant responding across seasons and more finely illustrate how these changes contribute to the overall effect on the DRL seen in Figure 5. Figure 6 contains a set of cumulative, single-session records for an individual bird across four seasons and illustrates the changes in response patterns across the year. Although the individual bird represented in Figure 6 exhibited the most extreme seasonal changes in responding, response records produced by other birds followed the same general seasonal pattern. The slope of the graph is an indicator of the rate of responding, with the pen resetting to zero for each 100 responses emitted during the session. The diagonal hash marks indicate when the reinforcers were acquired. During the winter session, rates of responding were low and 17 reinforcers were earned in the 30 min session. The pattern is similar during the summer session, although the response rate and number of reinforcers were slightly higher. Both of these records show steady, paced responding typical for the DRL schedule. During the spring session, however, response rate was extremely high; there were more than 700 responses emitted without a single reinforcer obtained in the 30-minute session. The fall session record is similar to spring although the rate is about half that of spring, with only 2 reinforcers obtained.

Figure 7 summarizes how the entire group of DRL birds performed within the 30-minute sessions during each season. Early session response rates are appropriately low during the non-migratory seasons of winter and summer. In the two migratory periods, response rates are higher. Regardless of season, responding decreased following the first 3-minute epoch in the session, particularly during spring, summer and fall, and this pattern of change did not differ throughout the week. During winter, summer and fall, this decreasing trend in response rate did not produce a concomitant decrease in reinforcement. However, during spring, the within session response decay actually led to a linear increase (p < 0.05) in reinforcement across the session; performance thus "improved" as response rates declined. Despite the "improved" responding during the end of each spring session, performance at the beginning of the subsequent session returned to previous, early-session levels.

Issues in premature responding on the DRL can be further qualified by examining the inter-response time intervals (IRTs) distribution, an analysis of the time intervals a bird waited before emitting a response. Figure 8 shows the relative frequency distribution of IRTs using a bin size of 2 seconds averaged across birds in each season. The proportion of reinforced IRTs, those which occur after the 20 second delay, is shown to the right of the dashed line. In a DRL 20-s schedule, the highest proportion of responses should ideally be in IRT intervals > 20 s, whereas a high proportion of responses in IRT intervals < 20 s indicates premature responding. On average, the smallest proportion of reinforced IRTs occurred during spring, followed by fall, then summer; the greatest proportion of reinforced IRTs occurred in winter. IRT distributions for DRL schedules were bimodal with the modes at the shortest (non-reinforced) and largest (reinforced) bins. Although the temporal placement of the modes differs subtly in each season, there was no definitive trend toward short duration IRTs in the two migratory seasons relative to the non-migratory seasons.

### Sleep Deprivation

**Figure 9:**
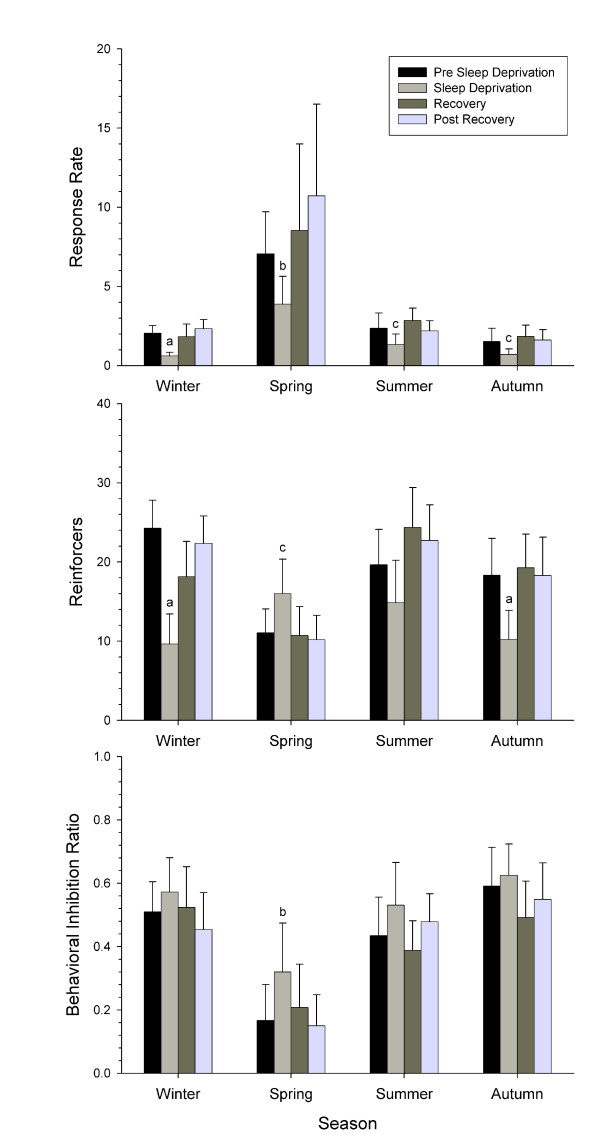
**48 hour sleep deprivation with the DRL birds**. Top shows response rate (responses/minute), center shows number of reinforcers acquired, and bottom shows the behavioral inhibition ratio (reinforcers/responses). Data shown are organized by season, Sleep deprivation decreased response rate regardless of season. The letters *a*, *b*, and *c *indicate statistical differences between DRL performance during the sleep deprivation compared to Pre Sleep Deprivation. Pre Sleep Deprivation performance did not differ statistically from Recovery or Post Recovery for any season, although average response rates during Post Recovery were about 25% (Winter) or 50% (Spring) higher than during Pre Sleep Deprivation. *N *= 15. Error bars represent half of the 95% CI.

Figure 9 shows the effects of sleep deprivation on performance data by season. The expected migratory increase in response rate is evident for spring but not fall, consistent with the fact that the birds were no longer actively migrating. Sleep-deprived birds tested on the DRL in fall were selected from weeks that did not include those that were used to assess general seasonal performance on the DRL. Regardless of season, the sleep deprivation manipulation affected response rate across conditions (p < 0.05), as well as the number of reinforcers obtained across conditions (p < 0.05). During the sleep deprivation period, response rate was significantly lower relative to pre sleep deprivation baseline during both winter and spring (post-hoc t, familywise p < 0.05). In winter, this resulted in a significant reduction in the number of reinforcers obtained (post-hoc t, familywise p < 0.05). However, in spring, the reduction in response rate produced a significant increase in reinforcers (post-hoc t, familywise p < 0.05). As a result, spring was the only season during which the sleep deprivation experiment produced a significant effect on the behavioral inhibition ratio (post-hoc t, familywise p < 0.05): In this case, we observed a significant increase in inhibition during the actual sleep deprivation relative to baseline (post-hoc t, familywise p < 0.05). For all dependent measures and seasons, recovery levels and post recovery levels did not differ statistically from baseline or from each other.

## Discussion

We previously reported that migratory White-crowned Sparrows housed under 12:12 light/dark reduced sleep during the migratory period by up to 60% relative to the non-migratory period [1]. Here we have also observed a reduction in the duration of total sleep when the two migratory seasons are compared to two non-migratory seasons; However, when considering season without regard to migratory status, only sleep duration in winter is significantly different from each of the other three seasons. As in mammals and other avian species, the changes in the daily duration of sleep are impacted by the seasonal changes in day length [16-18]. In the non-migratory winter season, the major sleep period was confined to the 14.5 hour dark period and sleep consumed 36% of the 24 hour period. In summer, however, when the nocturnal dark period was a mere 2.5 hours, sleep occupied only 14% of the 24 hour period, an amount comparable to that observed in each migratory period. If the White-crowned Sparrow routinely alters sleep duration across the calendar year, the amount of sleep lost during the migratory period may not, in fact, be physiologically significant.

The duration of sleep, in both birds and mammals, varies considerably as a result of environmental demands [19-22], and it is therefore difficult to define an optimal sleep amount. It is possible, however, that the duration of sleep observed here in the summer and winter represent the maximum and minimum sleep obtained in a day for the White-crowned Sparrow, respectively. The amount of sleep observed here during non-migratory winter on a photoperiod of 9.5:14.5 LD (36.6 ± 2.2%) was quite similar to that observed in our previous study during non-migratory late summer under conditions of 12:12 LD (33.8 ± 3.7%). Thus despite the fact that the dark period in our previous study was 2.5 hours longer than that used here, sleep duration was essentially unchanged, suggesting that about 36% of the 24 hour period may represent maximal sleep duration that these birds can achieve. In non-migratory summer, sleep is initiated after ~19 hours of wakefulness, with a considerable percentage of this sleep occurring during the light period. The onset of sleep during the light period, a time when sleep is ecologically suboptimal, suggests that selective pressure for maintaining at least 5 hours of sleep in each 24 h is stronger than that for competing behaviors.

Given that sleep duration in non-migratory summer is nearly identical to that observed in each of the migratory periods, the most striking difference between migratory and non-migratory sleep is the change from discrete day-night temporal organization to almost complete temporal fragmentation, an effect which, at least in the seasonal mammals, appears to be mediated by endogenous seasonal mechanisms rather than light duration [23]. Non-migratory days were organized in a clear biphasic fashion with maximal sleep occurring during the dark and maximal wakefulness during the day. A period of consolidated sleep occupying all of the dark phase was identifiable for both winter and summer, with 92% and 85% of all sleep occurring during this time in winter and summer, respectively. In contrast, during the migratory seasons, sleep and wakefulness were no longer temporally organized by the light-dark cycle. Although sleep duration in summer and the two migratory periods is comparable, whether this temporally fragmented sleep is as restorative as the consolidated sleep observed in summer is unclear. We were not able to reliably measure slow-wave activity (SWA), an EEG index of sleep intensity, so could not determine if it was preserved across seasons. It is conceivable, however, that migratory birds are capable of maintaining SWA over the course of a 24 hour day in those brief bouts of sleep and drowsiness which occur throughout the day. There is indeed empirical support for the idea that sleep intensity can be preserved in this manner [4,24,25]. In humans and many other mammals optimal sleep often requires an extended monophasic sleep period appropriately tuned to the circadian cycle [26]. However, this does not appear to be true across phylogeny or, rather, it may not be the case when external stimuli, such as food availability, temperature, or reproductive or migratory pressures demand organismic flexibility [19-21,27].

We previously demonstrated that when trained on an operant task used to assess learning and performance [28], neither the percentage of errors on sequence acquisition nor the ability to execute a previously learned response changed as a function of migratory status [1]; however, we did observe significant increase in operant responding rates during migration, an effect which is in direct contrast to the decreased operant responding associated with enforced sleep deprivation [1,29]. Similarly, on the DRL-20s task used here, rates of responding increased during the migratory period, despite the fact that such increases proved maladaptive. The average response rates on the DRL during the two migratory seasons were two times higher than response rates required to maximize reinforcement acquisition, and significantly higher than those observed in non-migratory winter and summer. Within individual DRL sessions, the most notable differences were apparent in the distribution of interresponse times (IRTs), indicating that when migratory birds did mistakenly respond during an interval when a response was not appropriate, they were much more likely to be near the beginning of the 'waiting' interval. Although it could be argued that the DRL is not an ecologically salient reinforcement schedule for migratory birds because waiting around, at least in the wild, is potentially maladaptive. However, in a highly controlled experimental environment, one might expect the behavior of these birds to come under control of the task contingencies. This clearly occurred during the winter and summer, but during the spring and fall when birds were actively migrating, the ability to execute this well-learned behavior was impaired.

The DRL is often used to measure aspects of impulsivity, a construct covering a wide range of behaviors considered to be unduly risky or inappropriate to the context. In humans, exogenously induced sleep deprivation has been shown to increase real-world risk taking behaviors [30-32], and to affect performance on laboratory tasks specifically designed to measure impulsivity [33-36]. It appears unlikely however, that migratory sleep loss plays a dominant role in migration-associated impulsivity. In summer, although total sleep duration was as short as that observed in the migratory seasons, birds maintained the ability to perform optimally on the DRL. Moreover, regardless of the degree of behavioral inhibition observed in each season, exogenously enforced sleep deprivation consistently had the effect of reducing response rates. If decreased sleep were mediating the increased response rates, we would have expected increased responding in summer or following sleep deprivation. Since sleep deprivation and migratory sleep loss have opposite effects on operant performance, it appears that migratory sleep loss might represent a change in sleep requirement, rather than a state of sleep deprivation, per se. It is conceivable that the temporal fragmentation of migratory sleep plays a role in the migration-specific loss of behavioral inhibition. Although sleep fragmentation and sleep restriction affect cognitive function [37], the differential effects of each are difficult to tease apart, and as such, a specific role for sleep fragmentation in impulsivity has not been characterized. Whether an inability to inhibit pecking is related to a general failure of inhibition, a distorted sense of time, inattention to salient cues, decreased sleep, or some other underlying mechanism is not entirely clear.

Although the neurobiological changes that underpin migration are largely unexplored, it is interesting that similar sleep abnormalities as well as impulsive behavior are prominent features of several psychiatric disorders. In fact, sleep reduction and increased impulsivity are arguably the most relevant symptoms of bipolar mania [38], and in many bipolar patients a seasonal presentation of symptoms is common [39-42]. It is possible that uncovering the mechanisms involved in the disrupted sleep and impulsive phenotype of the migratory bird has the potential to provide insight into bipolar disorder and perhaps other disorders associated with increased impulsivity and sleep disturbance, such as attention-deficit hyperactivity disorder [43].

### Limitations

Although we observed dramatic changes in sleep amounts and organization of sleep across seasons, sleep patterns in captive birds may not reflect those of birds in the wild. For example, sparrows in this study were not able to engage in prolonged nocturnal flights during the migratory season and instead spent their active time at night hopping around their cages, whirring their wings and/or trying to initiate flight. We could not determine, for example, if during nocturnal flight birds might engage in compensatory behavior such as unihemspheric sleep [44-46], or whether sleep amounts might be different in captivity due to physical restriction and decreased environmental stimulation. For example, the sloth sleeps many more hours when recorded in captivity than in the wild [47]. Nevertheless, these data demonstrate that sleep in the White-crowned Sparrow is significantly affected by both duration of the photoperiod and migratory status, although the magnitude of these effects may be different in captive birds vs. those in their natural habitat.

It is possible that changes in body weight played a role in the differences in DRL performance at different times of the year. For the DRL birds, attempts were made to minimize food restriction while still establishing food as an effective reinforcer by removing food from home cages 3 hours before operant sessions. As a result, body weights fluctuated considerably in our birds. Changes in body weight, however small, lead to changes in relative food restriction, which are known to affect operant behavior in birds [48]. However, given that migratory birds have higher caloric needs during the migratory seasons, it would seem that the food reinforcer would have more value during the migratory period. We would then expect to see improved performance as a function of migration, but this was clearly not the case.

## Conclusions

The amount of sleep required by the White-crowned Sparrow appears to be highly dynamic and contingent upon prevailing environmental conditions as well as physiological (migratory) status of the birds. Here we have observed marked changes in sleep duration as a function of seasonal day length, with sleep duration in summer occupying only 14% of the 24 hour day, a proportion comparable to that observed during both of the two migratory seasons analyzed. The most striking difference between migratory and non-migratory season sleep was therefore not related to the change in sleep duration, but rather to the loss of temporal organization of migratory sleep; during migratory periods, birds lost their usual diurnal pattern of wakefulness and sleep. Performance on a task used to assess behavioral inhibition was also notably changed as a function of migratory status. Although birds effectively learned to execute a delay period on the DRL-20s during the two non-migratory periods, they were not capable of sustaining this behavior in either the fall or spring migratory periods. Response rates were significantly elevated during migration, despite the fact that such behavior proved maladaptive. Since neither exogenously enforced sleep deprivation, or the short sleep duration observed in summer resulted in increased responding and impaired behavioral inhibition on the DRL task used, it appears that sleep loss is not mediating the impulsivity observed during migration. The observed seasonal changes in sleep patterns and behavior in the migratory sparrow raise interesting questions regarding possible correlates of these behaviors in other species, including humans.

## Competing interests

Over the past 2 years, Dr. Benca has served as a consultant for Actelion, Merck, Sanofi-aventis and Schering Plough.

## Authors' contributions

SGJ wrote most of the manuscript and coordinated the collection of sleep data; EMP coordinated the operant studies, analyzed operant data, generated relevant figures and helped draft the manuscript. WHO analyzed the sleep EEG data, generated all of the relevant figures, and helped draft the manuscript; CTH was responsible for the collection and scoring of all EEG data. RMB coordinated the development of the study, provided financial support for the project, and helped draft the manuscript. All authors read and approved the final manuscript.
